# 2-Methyl-*N*-(2-methyl­benzo­yl)benzene­sulfonamide

**DOI:** 10.1107/S1600536810026735

**Published:** 2010-07-10

**Authors:** P. A. Suchetan, B. Thimme Gowda, Sabine Foro, Hartmut Fuess

**Affiliations:** a Department of Chemistry, Mangalore University, Mangalagangotri 574 199, Mangalore, India; b Institute of Materials Science, Darmstadt University of Technology, Petersenstrasse 23, D-64287 Darmstadt, Germany

## Abstract

In the title compound, C_15_H_15_NO_3_S, the 2-methyl­phenyl ring bonded to the sulfonyl group is disordered with site-occupation factors of 0.75:0.25. The dihedral angles between the two aromatic rings are 67.6 (1) and 69.2 (1)° for the major and the minor occupied sites, respectively. In the crystal, mol­ecules are linked into centrosymmetric dimers by pairs of N—H⋯O hydrogen bonds.

## Related literature

For background literature and similar structures, see: Gowda *et al.* (2010**a* ▶,b* ▶); Suchetan *et al.* (2010 ▶).
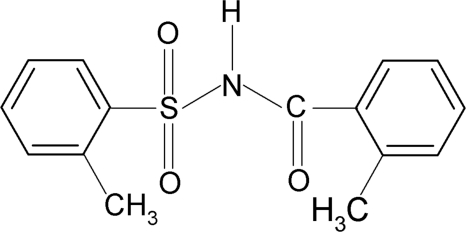


## Experimental

### 

#### Crystal data

C_15_H_15_NO_3_S
*M* *_r_* = 289.34
Monoclinic, 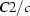

*a* = 13.997 (1) Å
*b* = 14.165 (1) Å
*c* = 14.395 (2) Å
β = 96.955 (8)°
*V* = 2833.1 (5) Å^3^
*Z* = 8
Mo *K*α radiation
μ = 0.24 mm^−1^
*T* = 299 K
0.40 × 0.36 × 0.34 mm

#### Data collection

Oxford Diffraction Xcalibur diffractometer with a Sapphire CCD detector
Absorption correction: multi-scan (*CrysAlis RED*; Oxford Diffraction, 2009 ▶) *T*_min_ = 0.912, *T*_max_ = 0.925
5920 measured reflections
2895 independent reflections
2281 reflections with *I* > 2σ(*I*)
*R*_int_ = 0.015

#### Refinement

*R*[*F* ^2^ > 2σ(*F* ^2^)] = 0.037
*wR*(*F* ^2^) = 0.114
*S* = 1.12
2895 reflections
251 parameters
9 restraints
H atoms treated by a mixture of independent and constrained refinement
Δρ_max_ = 0.30 e Å^−3^
Δρ_min_ = −0.26 e Å^−3^

### 

Data collection: *CrysAlis CCD* (Oxford Diffraction, 2009 ▶); cell refinement: *CrysAlis RED* (Oxford Diffraction, 2009 ▶); data reduction: *CrysAlis RED*; program(s) used to solve structure: *SHELXS97* (Sheldrick, 2008 ▶); program(s) used to refine structure: *SHELXL97* (Sheldrick, 2008 ▶); molecular graphics: *PLATON* (Spek, 2009 ▶); software used to prepare material for publication: *SHELXL97*.

## Supplementary Material

Crystal structure: contains datablocks I, global. DOI: 10.1107/S1600536810026735/bt5290sup1.cif

Structure factors: contains datablocks I. DOI: 10.1107/S1600536810026735/bt5290Isup2.hkl

Additional supplementary materials:  crystallographic information; 3D view; checkCIF report

## Figures and Tables

**Table 1:** Hydrogen-bond geometry (Å, °)

*D*—H⋯*A*	*D*—H	H⋯*A*	*D*⋯*A*	*D*—H⋯*A*
N1—H1*N*⋯O1^i^	0.86 (1)	2.10 (1)	2.9531 (17)	172 (2)

Symmetry code: (i) 
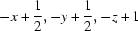
.

**Fig. 1.**
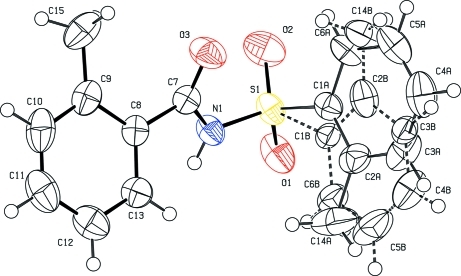
Molecular structure of (I), showing the atom labeling and displacement ellipsoids drawn at the 50% probability level. Both disorder components are shown. The minor disorder component is shown with dashed bonds.

**Fig. 2.**
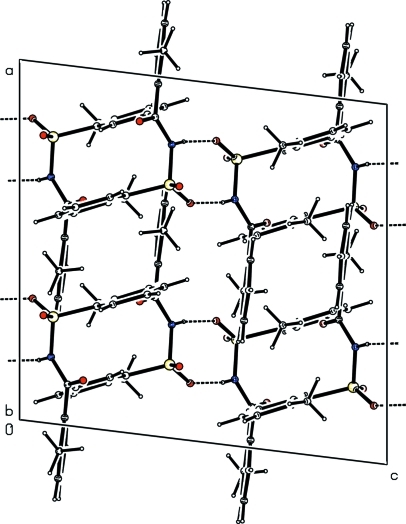
Molecular packing of (I) with hydrogen bonding shown as dashed lines. For clarity the minor occupied disorder component was omitted.

## supplementary crystallographic information

### Comment

Diaryl acylsulfonamides are known as potent antitumor agents against a broad spectrum of human tumor xenografts in nude mice. As part of a study of the effect of ring and the side chain substituents on the crystal structures of *N*-aromatic sulfonamides (Gowda *et al.*, 2010*a,b*; Suchetan *et al.*, 2010), in the present work, the structure of 2-methyl-*N*-(2-methylbenzoyl)benzenesulfonamide (I) has been determined (Fig.1). The conformation of the N—H bond in the S—NH—C═O segment of the structure is *anti* to the C═O bond, similar to that observed in 2-methyl-*N*-(3-methylbenzoyl)benzenesulfonamide (II) (Gowda *et al.*, 2010*a*), 2-methyl-*N*-(4-methylbenzoyl)- benzenesulfonamide (III) (Gowda *et al.*, 2010*b*) and 2-chloro-*N*-(2-chlorobenzoyl)benzenesulfonamide (IV) (Suchetan *et al.*, 2010).

The conformation of the C=O bond is *syn* to the *ortho*-methyl group in the benzoyl ring, similar to that observed between the C=O bond and the *ortho*-Cl in (IV), but contrary to the *anti* conformation observed between the C=O bond and the *meta*-methyl group in (II).

The molecules are twisted at the *S* atom with the torsional angles of 57.8 (2)° (major component) and 78.6 (2)° (minor component), compared to the values of -66.2 (3)° in (II), -53.1 (2)° and 61.2 (2)° in the two molecules of (III), and 66.5 (3)° in (IV),

The dihedral angles between the sulfonyl benzene ring and the S—NH—C═O segment are 82.7 (1)° (major component) and 85.1 (3)° (minor component), compared to the values of 83.1 (1) in (II), 86.0 (1)° and 87.9 (1)° in the two molecules of (III), and 86.9 (1) in (IV)

The dihedral angle between the sulfonyl and the benzoyl benzene rings are 67.6 (1)° and 69.2 (1)° in the major and the minor components, respectively, compared to the values of 74.8 (1) in (II), 88.1 (1)° (molecule 1) and 83.5 (1)° (molecule 2) of (III), and 76.9 (1) in (IV).

The molecules are linked into chains by N—H···O(S) hydrogen bonds (Table 1 and Fig. 2).

### Experimental

The title compound was prepared by refluxing a mixture of 2-methylbenzoic acid, 2-methylbenzenesulfonamide and phosphorous oxy chloride for 5 h on a water bath. The reaction mixture was cooled and poured into ice cold water. The resulting solid, 2-methyl-*N*-(2-methylbenzoyl)benzenesulfonamide, was separated, washed thoroughly with water and then dissolved in sodium bicarbonate solution. The compound was later reprecipitated by acidifying the filtered solution with dilute HCl. The filtered and dried compound was recrystallized to the constant melting point.

Prism like colourless single crystals of the title compound were grown by slow evaporation of its toluene solution at room temperature.

### Refinement

The H atom of the NH group was located in a difference map and   restrained to N—H = 0.86 (1) %A. The other H atoms were positioned with idealized geometry using a riding model with C—H = 0.93–0.96 Å. All H atoms were refined with isotropic displacement parameters  set to 1.2 times of the *U*_eq_ of the parent atom.

The methylphenyl ring with atoms C1, C2, C3, C4, C5, C6 and C14 is disordered over two sites related by a twofold rotation. The corresponding site-occupation factors were fixed to 0.75:0.25 and their corresponding bond distances in the disordered groups were restrained to be equal.

### Crystal data

**Table:**

C_15_H_15_NO_3_S	*F*(000) = 1216
*M**_r_* = 289.34	*D*_x_ = 1.357 Mg m^−^^3^
Monoclinic, *C*2/*c*	Mo *K*α radiation, λ = 0.71073 Å
Hall symbol: -C 2yc	Cell parameters from 2699 reflections
*a* = 13.997 (1) Å	θ = 2.6–27.8°
*b* = 14.165 (1) Å	µ = 0.24 mm^−^^1^
*c* = 14.395 (2) Å	*T* = 299 K
β = 96.955 (8)°	Prism, colourless
*V* = 2833.1 (5) Å^3^	0.40 × 0.36 × 0.34 mm
*Z* = 8	

### Data collection

**Table:**

Oxford Diffraction Xcalibur diffractometer with a Sapphire CCD detector	2895 independent reflections
Radiation source: fine-focus sealed tube	2281 reflections with *I* > 2σ(*I*)
graphite	*R*_int_ = 0.015
Rotation method data acquisition using ω and phi scans	θ_max_ = 26.4°, θ_min_ = 2.6°
Absorption correction: multi-scan (*CrysAlis RED*; Oxford Diffraction, 2009)	*h* = −14→17
*T*_min_ = 0.912, *T*_max_ = 0.925	*k* = −17→12
5920 measured reflections	*l* = −17→17

### Refinement

**Table:**

Refinement on *F*^2^	Primary atom site location: structure-invariant direct methods
Least-squares matrix: full	Secondary atom site location: difference Fourier map
*R*[*F*^2^ > 2σ(*F*^2^)] = 0.037	Hydrogen site location: inferred from neighbouring sites
*wR*(*F*^2^) = 0.114	H atoms treated by a mixture of independent and constrained refinement
*S* = 1.12	*w* = 1/[σ^2^(*F*_o_^2^) + (0.0707*P*)^2^] where *P* = (*F*_o_^2^ + 2*F*_c_^2^)/3
2895 reflections	(Δ/σ)_max_ < 0.001
251 parameters	Δρ_max_ = 0.30 e Å^−^^3^
9 restraints	Δρ_min_ = −0.26 e Å^−^^3^

### Special details

**Table:**

Experimental. CrysAlis RED (Oxford Diffraction, 2009) Empirical absorption correction using spherical harmonics, implemented in SCALE3 ABSPACK scaling algorithm.
Geometry. All e.s.d.'s (except the e.s.d. in the dihedral angle between two l.s. planes) are estimated using the full covariance matrix. The cell e.s.d.'s are taken into account individually in the estimation of e.s.d.'s in distances, angles and torsion angles; correlations between e.s.d.'s in cell parameters are only used when they are defined by crystal symmetry. An approximate (isotropic) treatment of cell e.s.d.'s is used for estimating e.s.d.'s involving l.s. planes.
Refinement. Refinement of *F*^2^ against ALL reflections. The weighted *R*-factor *wR* and goodness of fit *S* are based on *F*^2^, conventional *R*-factors *R* are based on *F*, with *F* set to zero for negative *F*^2^. The threshold expression of *F*^2^ > σ(*F*^2^) is used only for calculating *R*-factors(gt) *etc*. and is not relevant to the choice of reflections for refinement. *R*-factors based on *F*^2^ are statistically about twice as large as those based on *F*, and *R*- factors based on ALL data will be even larger.

### Fractional atomic coordinates and isotropic or equivalent isotropic displacement parameters (Å^2^)

**Table:**

	*x*	*y*	*z*	*U*_iso_*/*U*_eq_	Occ. (<1)
C1A	0.1586 (2)	0.3862 (2)	0.2895 (2)	0.0392 (7)	0.75
C1B	0.1568 (6)	0.3398 (6)	0.2942 (6)	0.0354 (18)	0.25
C2A	0.13372 (16)	0.3021 (2)	0.2415 (2)	0.0506 (6)	0.75
C2B	0.1299 (5)	0.3961 (6)	0.2180 (5)	0.0528 (18)	0.25
C3A	0.0925 (6)	0.3103 (4)	0.1473 (4)	0.0680 (17)	0.75
H3A	0.0726	0.2567	0.1131	0.082*	0.75
C3B	0.0951 (16)	0.3438 (9)	0.1388 (10)	0.041 (3)	0.25
H3B	0.0742	0.3779	0.0849	0.050*	0.25
C4A	0.0817 (2)	0.3986 (3)	0.1061 (2)	0.0712 (8)	0.75
H4A	0.0543	0.4029	0.0441	0.085*	0.75
C4B	0.0886 (6)	0.2496 (8)	0.1325 (6)	0.066 (2)	0.25
H4B	0.0648	0.2220	0.0758	0.079*	0.25
C5A	0.1093 (2)	0.4781 (2)	0.1523 (2)	0.0650 (8)	0.75
H5A	0.1024	0.5363	0.1223	0.078*	0.75
C5B	0.1164 (7)	0.1931 (7)	0.2084 (7)	0.079 (3)	0.25
H5B	0.1133	0.1276	0.2056	0.095*	0.25
C6A	0.1483 (3)	0.4725 (3)	0.2459 (3)	0.0505 (8)	0.75
H6A	0.1675	0.5272	0.2788	0.061*	0.75
C6B	0.1493 (10)	0.2411 (8)	0.2890 (9)	0.050 (3)	0.25
H6B	0.1675	0.2062	0.3430	0.060*	0.25
C7	0.38609 (11)	0.39162 (11)	0.36903 (10)	0.0396 (3)	
C8	0.47917 (10)	0.33980 (11)	0.37729 (10)	0.0390 (3)	
C9	0.56650 (11)	0.38885 (13)	0.38842 (11)	0.0496 (4)	
C10	0.65018 (12)	0.33451 (18)	0.39523 (14)	0.0675 (6)	
H10	0.7094	0.3651	0.4045	0.081*	
C11	0.64890 (13)	0.23848 (17)	0.38881 (14)	0.0712 (6)	
H11	0.7064	0.2050	0.3933	0.085*	
C12	0.56284 (14)	0.19138 (15)	0.37571 (13)	0.0614 (5)	
H12	0.5614	0.1260	0.3699	0.074*	
C13	0.47861 (12)	0.24202 (12)	0.37119 (11)	0.0479 (4)	
H13	0.4201	0.2101	0.3639	0.057*	
C14A	0.1486 (5)	0.2049 (3)	0.2834 (5)	0.0866 (18)	0.75
H14A	0.1053	0.1957	0.3295	0.104*	0.75
H14B	0.2138	0.1989	0.3124	0.104*	0.75
H14C	0.1362	0.1583	0.2351	0.104*	0.75
C14B	0.1351 (11)	0.4964 (9)	0.2142 (8)	0.062 (3)	0.25
H14D	0.1919	0.5176	0.2526	0.074*	0.25
H14E	0.0792	0.5231	0.2368	0.074*	0.25
H14F	0.1376	0.5160	0.1508	0.074*	0.25
C15	0.57390 (14)	0.49465 (14)	0.39237 (14)	0.0687 (6)	
H15C	0.6380	0.5125	0.4176	0.082*	
H15B	0.5288	0.5190	0.4316	0.082*	
H15A	0.5596	0.5201	0.3304	0.082*	
N1	0.31537 (9)	0.34845 (10)	0.41437 (9)	0.0439 (3)	
H1N	0.3279 (12)	0.2983 (8)	0.4467 (11)	0.053*	
O1	0.15903 (8)	0.32012 (10)	0.46446 (9)	0.0645 (4)	
O2	0.20209 (10)	0.48187 (10)	0.43476 (10)	0.0743 (4)	
O3	0.36933 (9)	0.46426 (9)	0.32707 (9)	0.0609 (4)	
S1	0.20355 (3)	0.38584 (3)	0.40857 (3)	0.04381 (16)	

### Atomic displacement parameters (Å^2^)

**Table:**

	*U*^11^	*U*^22^	*U*^33^	*U*^12^	*U*^13^	*U*^23^
C1A	0.0344 (11)	0.0406 (16)	0.0446 (15)	0.0054 (14)	0.0126 (10)	0.0021 (15)
C1B	0.028 (3)	0.032 (5)	0.046 (4)	0.001 (4)	0.004 (3)	−0.011 (4)
C2A	0.0416 (13)	0.0475 (15)	0.0645 (17)	−0.0073 (11)	0.0139 (12)	−0.0012 (16)
C2B	0.045 (4)	0.076 (6)	0.038 (4)	0.008 (3)	0.005 (3)	0.002 (4)
C3A	0.0522 (19)	0.075 (5)	0.078 (3)	−0.018 (4)	0.015 (2)	−0.024 (3)
C3B	0.043 (5)	0.041 (8)	0.038 (5)	−0.008 (7)	−0.003 (4)	0.002 (5)
C4A	0.0545 (16)	0.110 (3)	0.0481 (16)	0.0103 (18)	0.0022 (13)	−0.0010 (18)
C4B	0.058 (5)	0.083 (7)	0.057 (5)	−0.022 (5)	0.011 (4)	−0.012 (5)
C5A	0.0680 (18)	0.076 (2)	0.0507 (15)	0.0261 (15)	0.0078 (13)	0.0176 (16)
C5B	0.076 (6)	0.065 (6)	0.098 (7)	−0.018 (5)	0.014 (5)	−0.041 (6)
C6A	0.0564 (17)	0.049 (2)	0.047 (2)	0.0174 (15)	0.0092 (15)	0.0061 (15)
C6B	0.049 (4)	0.046 (8)	0.054 (5)	−0.005 (6)	0.004 (3)	−0.012 (6)
C7	0.0404 (8)	0.0437 (9)	0.0353 (7)	−0.0031 (6)	0.0067 (6)	0.0002 (7)
C8	0.0363 (8)	0.0497 (9)	0.0318 (7)	−0.0026 (6)	0.0077 (6)	0.0028 (7)
C9	0.0449 (9)	0.0658 (11)	0.0392 (8)	−0.0119 (8)	0.0096 (7)	0.0006 (8)
C10	0.0349 (9)	0.1043 (17)	0.0642 (11)	−0.0074 (9)	0.0089 (8)	0.0060 (11)
C11	0.0480 (12)	0.0952 (17)	0.0733 (13)	0.0213 (11)	0.0186 (9)	0.0147 (12)
C12	0.0604 (11)	0.0623 (12)	0.0648 (11)	0.0151 (9)	0.0214 (9)	0.0044 (9)
C13	0.0445 (9)	0.0491 (9)	0.0518 (9)	−0.0002 (7)	0.0134 (7)	0.0028 (8)
C14A	0.103 (3)	0.043 (3)	0.118 (4)	−0.016 (3)	0.029 (3)	−0.001 (3)
C14B	0.081 (8)	0.045 (7)	0.058 (9)	0.014 (5)	0.004 (7)	0.007 (6)
C15	0.0678 (12)	0.0737 (14)	0.0661 (12)	−0.0303 (10)	0.0151 (9)	−0.0087 (10)
N1	0.0364 (7)	0.0493 (8)	0.0471 (7)	0.0055 (6)	0.0094 (6)	0.0136 (6)
O1	0.0443 (7)	0.0843 (9)	0.0688 (8)	0.0123 (6)	0.0224 (6)	0.0321 (7)
O2	0.0819 (10)	0.0615 (9)	0.0828 (10)	0.0224 (7)	0.0240 (8)	−0.0093 (7)
O3	0.0606 (8)	0.0533 (7)	0.0711 (8)	0.0053 (6)	0.0179 (6)	0.0228 (6)
S1	0.0401 (2)	0.0535 (3)	0.0392 (2)	0.01159 (17)	0.01069 (16)	0.00625 (17)

### Geometric parameters (Å, °)

**Table:**

C1A—C6A	1.374 (4)	C7—C8	1.488 (2)
C1A—C2A	1.400 (3)	C8—C13	1.388 (2)
C1A—S1	1.753 (3)	C8—C9	1.398 (2)
C1B—C2B	1.372 (10)	C9—C10	1.395 (3)
C1B—C6B	1.403 (10)	C9—C15	1.503 (3)
C1B—S1	1.817 (8)	C10—C11	1.363 (3)
C2A—C3A	1.412 (7)	C10—H10	0.9300
C2A—C14A	1.507 (5)	C11—C12	1.370 (3)
C2B—C3B	1.397 (13)	C11—H11	0.9300
C2B—C14B	1.423 (13)	C12—C13	1.375 (2)
C3A—C4A	1.384 (5)	C12—H12	0.9300
C3A—H3A	0.9300	C13—H13	0.9300
C3B—C4B	1.340 (13)	C14A—H14A	0.9600
C3B—H3B	0.9300	C14A—H14B	0.9600
C4A—C5A	1.341 (5)	C14A—H14C	0.9600
C4A—H4A	0.9300	C14B—H14D	0.9600
C4B—C5B	1.372 (12)	C14B—H14E	0.9600
C4B—H4B	0.9300	C14B—H14F	0.9600
C5A—C6A	1.393 (4)	C15—H15C	0.9600
C5A—H5A	0.9300	C15—H15B	0.9600
C5B—C6B	1.375 (13)	C15—H15A	0.9600
C5B—H5B	0.9300	N1—S1	1.6449 (13)
C6A—H6A	0.9300	N1—H1N	0.856 (9)
C6B—H6B	0.9300	O1—S1	1.4226 (12)
C7—O3	1.2018 (18)	O2—S1	1.4123 (14)
C7—N1	1.3915 (19)		
			
C6A—C1A—C2A	121.7 (3)	C9—C8—C7	120.59 (15)
C6A—C1A—S1	117.0 (3)	C10—C9—C8	116.66 (17)
C2A—C1A—S1	121.2 (2)	C10—C9—C15	119.61 (16)
C2B—C1B—C6B	121.7 (9)	C8—C9—C15	123.72 (16)
C2B—C1B—S1	123.3 (7)	C11—C10—C9	122.80 (17)
C6B—C1B—S1	115.0 (7)	C11—C10—H10	118.6
C1A—C2A—C3A	116.9 (3)	C9—C10—H10	118.6
C1A—C2A—C14A	124.4 (3)	C10—C11—C12	119.96 (17)
C3A—C2A—C14A	118.7 (4)	C10—C11—H11	120.0
C1B—C2B—C3B	112.2 (9)	C12—C11—H11	120.0
C1B—C2B—C14B	126.9 (8)	C11—C12—C13	119.16 (19)
C3B—C2B—C14B	120.9 (8)	C11—C12—H12	120.4
C4A—C3A—C2A	119.8 (5)	C13—C12—H12	120.4
C4A—C3A—H3A	120.1	C12—C13—C8	121.29 (17)
C2A—C3A—H3A	120.1	C12—C13—H13	119.4
C4B—C3B—C2B	126.7 (12)	C8—C13—H13	119.4
C4B—C3B—H3B	116.6	C2B—C14B—H14D	109.5
C2B—C3B—H3B	116.6	C2B—C14B—H14E	109.5
C5A—C4A—C3A	122.5 (4)	H14D—C14B—H14E	109.5
C5A—C4A—H4A	118.8	C2B—C14B—H14F	109.5
C3A—C4A—H4A	118.8	H14D—C14B—H14F	109.5
C3B—C4B—C5B	121.1 (9)	H14E—C14B—H14F	109.5
C3B—C4B—H4B	119.5	C9—C15—H15C	109.5
C5B—C4B—H4B	119.5	C9—C15—H15B	109.5
C4A—C5A—C6A	119.2 (3)	H15C—C15—H15B	109.5
C4A—C5A—H5A	120.4	C9—C15—H15A	109.5
C6A—C5A—H5A	120.4	H15C—C15—H15A	109.5
C4B—C5B—C6B	114.7 (9)	H15B—C15—H15A	109.5
C4B—C5B—H5B	122.6	C7—N1—S1	124.14 (11)
C6B—C5B—H5B	122.6	C7—N1—H1N	120.6 (12)
C1A—C6A—C5A	119.9 (4)	S1—N1—H1N	115.2 (12)
C1A—C6A—H6A	120.0	O2—S1—O1	117.24 (9)
C5A—C6A—H6A	120.0	O2—S1—N1	109.94 (8)
C5B—C6B—C1B	123.5 (10)	O1—S1—N1	103.88 (7)
C5B—C6B—H6B	118.3	O2—S1—C1A	104.07 (11)
C1B—C6B—H6B	118.3	O1—S1—C1A	115.14 (11)
O3—C7—N1	120.35 (14)	N1—S1—C1A	106.11 (10)
O3—C7—C8	125.34 (14)	O2—S1—C1B	124.9 (3)
N1—C7—C8	114.30 (13)	O1—S1—C1B	98.2 (3)
C13—C8—C9	120.09 (15)	N1—S1—C1B	99.6 (2)
C13—C8—C7	119.29 (13)		
			
C6A—C1A—C2A—C3A	−3.5 (6)	C8—C9—C10—C11	1.9 (3)
S1—C1A—C2A—C3A	176.1 (4)	C15—C9—C10—C11	−177.57 (19)
C6A—C1A—C2A—C14A	176.2 (4)	C9—C10—C11—C12	−0.4 (3)
S1—C1A—C2A—C14A	−4.2 (5)	C10—C11—C12—C13	−1.4 (3)
C6B—C1B—C2B—C3B	0.4 (16)	C11—C12—C13—C8	1.6 (3)
S1—C1B—C2B—C3B	−179.1 (12)	C9—C8—C13—C12	−0.1 (2)
C6B—C1B—C2B—C14B	−179.3 (12)	C7—C8—C13—C12	178.09 (14)
S1—C1B—C2B—C14B	1.2 (13)	O3—C7—N1—S1	5.0 (2)
C1A—C2A—C3A—C4A	2.3 (9)	C8—C7—N1—S1	−174.44 (10)
C14A—C2A—C3A—C4A	−177.4 (6)	C7—N1—S1—O2	−54.19 (15)
C1B—C2B—C3B—C4B	−1(3)	C7—N1—S1—O1	179.57 (13)
C14B—C2B—C3B—C4B	178.4 (18)	C7—N1—S1—C1A	57.78 (17)
C2A—C3A—C4A—C5A	0.1 (9)	C7—N1—S1—C1B	78.5 (3)
C2B—C3B—C4B—C5B	1(3)	C6A—C1A—S1—O2	10.4 (3)
C3A—C4A—C5A—C6A	−1.5 (6)	C2A—C1A—S1—O2	−169.2 (2)
C3B—C4B—C5B—C6B	1(2)	C6A—C1A—S1—O1	140.2 (2)
C2A—C1A—C6A—C5A	2.2 (5)	C2A—C1A—S1—O1	−39.5 (3)
S1—C1A—C6A—C5A	−177.4 (3)	C6A—C1A—S1—N1	−105.6 (3)
C4A—C5A—C6A—C1A	0.3 (5)	C2A—C1A—S1—N1	74.8 (2)
C4B—C5B—C6B—C1B	−1.7 (18)	C6A—C1A—S1—C1B	179.8 (10)
C2B—C1B—C6B—C5B	1.1 (18)	C2A—C1A—S1—C1B	0.2 (7)
S1—C1B—C6B—C5B	−179.4 (10)	C2B—C1B—S1—O2	11.0 (8)
O3—C7—C8—C13	−139.43 (17)	C6B—C1B—S1—O2	−168.6 (7)
N1—C7—C8—C13	39.97 (19)	C2B—C1B—S1—O1	142.7 (6)
O3—C7—C8—C9	38.8 (2)	C6B—C1B—S1—O1	−36.9 (8)
N1—C7—C8—C9	−141.84 (14)	C2B—C1B—S1—N1	−111.7 (7)
C13—C8—C9—C10	−1.6 (2)	C6B—C1B—S1—N1	68.8 (8)
C7—C8—C9—C10	−179.76 (14)	C2B—C1B—S1—C1A	−1.6 (5)
C13—C8—C9—C15	177.84 (16)	C6B—C1B—S1—C1A	178.9 (14)
C7—C8—C9—C15	−0.3 (2)		

### Hydrogen-bond geometry (Å, °)

**Table:**

*D*—H···*A*	*D*—H	H···*A*	*D*···*A*	*D*—H···*A*
N1—H1N···O1^i^	0.86 (1)	2.10 (1)	2.9531 (17)	172 (2)

Symmetry codes: (i) −*x*+1/2, −*y*+1/2, −*z*+1.
